# Methyl 4-(butyrylamino)-5-methyl-2-nitro­benzoate

**DOI:** 10.1107/S1600536808005576

**Published:** 2008-03-29

**Authors:** Lian-shan Yuan, Xiang Li, Cheng Yao

**Affiliations:** a Department of Applied Chemistry, College of Sciences, Nanjing University of Technolgy, Xinmofan Road No. 5 Nanjing, Nanjing 210009, People’s Republic of China

## Abstract

The title compound, C_13_H_16_N_2_O_5_, is useful as an inter­mediate in the field of agrochemicals. Intra­molecular C—H⋯O hydrogen bonds result in the formation of one six- and one five-membered nearly planar ring; the six-membered ring is also nearly coplanar with the adjacent benzene ring. In the crystal structure, inter­molecular C—H⋯O hydrogen bonds link the mol­ecules.

## Related literature

For related literature, see: Ries *et al.* (1993 ▶); Engeli *et al.* (2000 ▶); Kintscher *et al.* (2004 ▶); Goossens *et al.* (2003 ▶); Boustany *et al.* (2004 ▶). For bond-length data, see: Allen *et al.* (1987 ▶).
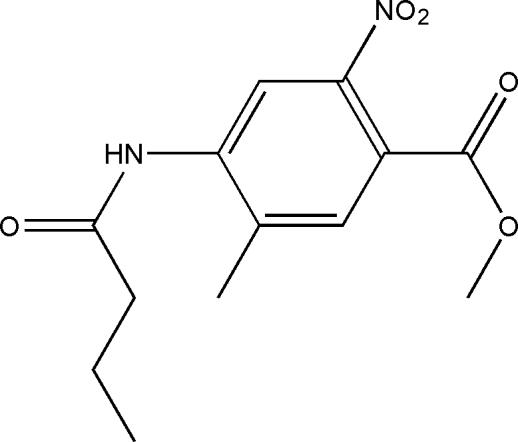


## Experimental

### 

#### Crystal data

C_13_H_16_N_2_O_5_
*M* *_r_* = 280.28
Triclinic, 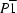

*a* = 7.6370 (15) Å
*b* = 8.7880 (18) Å
*c* = 11.329 (2) Å
α = 81.06 (3)°
β = 78.48 (3)°
γ = 68.39 (3)°
*V* = 689.9 (3) Å^3^
*Z* = 2
Mo *K*α radiation
μ = 0.10 mm^−1^
*T* = 294 (2) K
0.30 × 0.20 × 0.10 mm

#### Data collection

Enraf–Nonius CAD-4 diffractometer
Absorption correction: ψ scan (North *et al.*, 1968 ▶) *T*_min_ = 0.959, *T*_max_ = 0.980
2919 measured reflections
2704 independent reflections
1650 reflections with *I* > 2σ(*I*)
*R*_int_ = 0.032
3 standard reflections frequency: 120 min intensity decay: none

#### Refinement

*R*[*F* ^2^ > 2σ(*F* ^2^)] = 0.056
*wR*(*F* ^2^) = 0.161
*S* = 1.01
2704 reflections
181 parameters
H-atom parameters constrained
Δρ_max_ = 0.20 e Å^−3^
Δρ_min_ = −0.18 e Å^−3^

### 

Data collection: *CAD-4 Software* (Enraf–Nonius, 1989 ▶); cell refinement: *CAD-4 Software*; data reduction: *XCAD4* (Harms & Wocadlo, 1995 ▶); program(s) used to solve structure: *SHELXS97* (Sheldrick, 2008 ▶); program(s) used to refine structure: *SHELXL97* (Sheldrick, 2008 ▶); molecular graphics: *SHELXTL* (Sheldrick, 2008 ▶); software used to prepare material for publication: *SHELXTL*.

## Supplementary Material

Crystal structure: contains datablocks global, I. DOI: 10.1107/S1600536808005576/hk2408sup1.cif

Structure factors: contains datablocks I. DOI: 10.1107/S1600536808005576/hk2408Isup2.hkl

Additional supplementary materials:  crystallographic information; 3D view; checkCIF report

## Figures and Tables

**Table 1:** Hydrogen-bond geometry (Å, °)

*D*—H⋯*A*	*D*—H	H⋯*A*	*D*⋯*A*	*D*—H⋯*A*
C6—H6*A*⋯O1	0.93	2.20	2.809 (3)	122
C9—H9*A*⋯O4	0.93	2.41	2.734 (3)	100
C13—H13*A*⋯O1^i^	0.96	2.33	3.284 (4)	174

Symmetry code: (i) 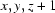.

**Fig. 1.**
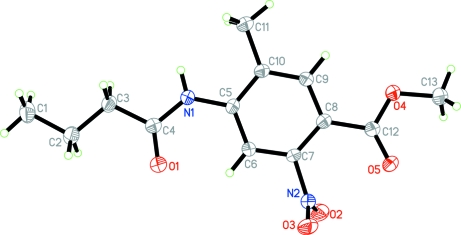
The molecular structure of the title molecule, with the atom-numbering scheme. Displacement ellipsoids are drawn at the 30% probability level.

**Fig. 2.**
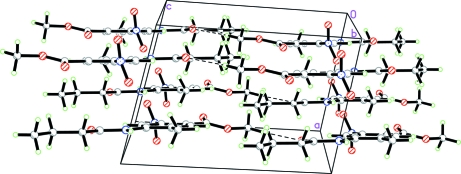
A packing diagram of (I). Hydrogen bonds are shown as dashed lines.

## supplementary crystallographic information

### Comment

The title compound, (I), is useful as an intermediate and agrochemicals. It is important as an intermediate for the preparation of telmisartan (Ries *et al.*, 1993), that can be used as a therapeutic tool for metabolic syndrome, including visceral obesity (Engeli *et al.*, 2000; Kintscher *et al.*, 2004; Goossens *et al.*, 2003; Boustany *et al.*, 2004). As part of our ongoing studies in this area, we report herein the synthesis and crystal structure of the title compound, (I).

In the molecule of (I) (Fig. 1), the ligand bond lengths and angles are within normal ranges (Allen *et al.*, 1987). The intramolecular C—H···O hydrogen bonds (Table 1) result in the formations of one six- and one five-membered nearly planar rings; B (N1/O1/C4—C6/H6A) and C (O4/C8/C9/C12/H9A). Ring A (C5—C10) is, of course, planar and the dihedral angles between them are A/B = 2.01 (3)°, A/C = 6.76 (3)° and B/C = 8.73 (2)°. So, rings A and B are also nearly co-planar.

In the crystal structure, intermolecular C—H···O hydrogen bonds (Table 1) link the molecules (Fig. 2), in which they seem to be effective in the stabilization of the structure.

### Experimental

For the preparation of the title compound, methyl 4-amino-3-methylbenzoate (8.25 g, 50 mmol) was acylated with butyryl chloride (50 mmol, 5.3 ml) in chlorobenzene at 373 K. The resulting amide was reacted with fuming nitric acid in sulfuric acid (60%) at 273 K. The reaction mixture was poured into ice-water. The residue was filtered and recrystallized from methylene chloride to give the title compound, (I), (yield; 10.8 g, 77%). Crystals suitable for X-ray analysis were obtained by slow evaporation of an ethanol solution.

### Refinement

H atoms were positioned geometrically, with N—H = 0.86 Å (for NH) and C—H = 0.93, 0.97 and 0.96 Å for aromatic, methine and methyl H, and constrained to ride on their parent atoms, with *U*_iso_(H) = *xU*_eq_(C,*N*), where *x* = 1.5 for methyl H, and *x* = 1.2 for all other H atoms.

### Crystal data

**Table:**

C_13_H_16_N_2_O_5_	*Z* = 2
*M**_r_* = 280.28	*F*_000_ = 296
Triclinic, *P*1	*D*_x_ = 1.349 Mg m^−^^3^
Hall symbol: -P 1	Mo *K*α radiation λ = 0.71073 Å
*a* = 7.6370 (15) Å	Cell parameters from 25 reflections
*b* = 8.7880 (18) Å	θ = 9–14º
*c* = 11.329 (2) Å	µ = 0.11 mm^−^^1^
α = 81.06 (3)º	*T* = 294 (2) K
β = 78.48 (3)º	Block, colorless
γ = 68.39 (3)º	0.30 × 0.20 × 0.10 mm
*V* = 689.9 (3) Å^3^	

### Data collection

**Table:**

Enraf–Nonius CAD-4 diffractometer	*R*_int_ = 0.032
Radiation source: fine-focus sealed tube	θ_max_ = 26.0º
Monochromator: graphite	θ_min_ = 1.8º
*T* = 294(2) K	*h* = −9→9
ω/2θ scans	*k* = −10→10
Absorption correction: ψ scan(North *et al.*, 1968)	*l* = 0→13
*T*_min_ = 0.959, *T*_max_ = 0.980	3 standard reflections
2919 measured reflections	every 120 min
2704 independent reflections	intensity decay: none
1650 reflections with *I* > 2σ(*I*)	

### Refinement

**Table:**

Refinement on *F*^2^	Secondary atom site location: difference Fourier map
Least-squares matrix: full	Hydrogen site location: inferred from neighbouring sites
*R*[*F*^2^ > 2σ(*F*^2^)] = 0.056	H-atom parameters constrained
*wR*(*F*^2^) = 0.161	*w* = 1/[σ^2^(*F*_o_^2^) + (0.06*P*)^2^ + 0.4*P*] where *P* = (*F*_o_^2^ + 2*F*_c_^2^)/3
*S* = 1.01	(Δ/σ)_max_ < 0.001
2704 reflections	Δρ_max_ = 0.20 e Å^−^^3^
181 parameters	Δρ_min_ = −0.18 e Å^−^^3^
Primary atom site location: structure-invariant direct methods	Extinction correction: none

### Special details

**Table:**

Geometry. All e.s.d.'s (except the e.s.d. in the dihedral angle between two l.s. planes) are estimated using the full covariance matrix. The cell e.s.d.'s are taken into account individually in the estimation of e.s.d.'s in distances, angles and torsion angles; correlations between e.s.d.'s in cell parameters are only used when they are defined by crystal symmetry. An approximate (isotropic) treatment of cell e.s.d.'s is used for estimating e.s.d.'s involving l.s. planes.
Refinement. Refinement of *F*^2^ against ALL reflections. The weighted *R*-factor *wR* and goodness of fit *S* are based on *F*^2^, conventional *R*-factors *R* are based on *F*, with *F* set to zero for negative *F*^2^. The threshold expression of *F*^2^ > σ(*F*^2^) is used only for calculating *R*-factors(gt) *etc*. and is not relevant to the choice of reflections for refinement. *R*-factors based on *F*^2^ are statistically about twice as large as those based on *F*, and *R*- factors based on ALL data will be even larger.

### Fractional atomic coordinates and isotropic or equivalent isotropic displacement parameters (Å^2^)

**Table:**

	*x*	*y*	*z*	*U*_iso_*/*U*_eq_	
N1	0.2769 (4)	−0.1219 (3)	−0.0125 (2)	0.0492 (6)	
H1A	0.3013	−0.2242	0.0119	0.059*	
O1	0.2083 (4)	0.0590 (3)	−0.1748 (2)	0.0845 (9)	
C1	0.2766 (5)	−0.3253 (4)	−0.3938 (3)	0.0681 (10)	
H1B	0.2607	−0.2909	−0.4769	0.102*	
H1C	0.1823	−0.3727	−0.3551	0.102*	
H1D	0.4016	−0.4056	−0.3901	0.102*	
N2	0.1750 (4)	0.4161 (3)	0.1056 (2)	0.0454 (6)	
O2	0.0124 (3)	0.5069 (2)	0.1365 (2)	0.0693 (7)	
C2	0.2531 (5)	−0.1773 (4)	−0.3296 (3)	0.0520 (8)	
H2A	0.3459	−0.1278	−0.3706	0.062*	
H2B	0.1270	−0.0962	−0.3341	0.062*	
O3	0.3005 (3)	0.4618 (3)	0.0453 (2)	0.0634 (7)	
C3	0.2792 (4)	−0.2244 (3)	−0.1980 (2)	0.0454 (7)	
H3A	0.1893	−0.2776	−0.1582	0.054*	
H3B	0.4066	−0.3034	−0.1941	0.054*	
O4	0.2265 (3)	0.1922 (2)	0.46212 (17)	0.0595 (6)	
C4	0.2510 (4)	−0.0805 (3)	−0.1302 (2)	0.0445 (7)	
O5	0.2355 (3)	0.4105 (2)	0.33631 (17)	0.0585 (6)	
C5	0.2695 (4)	−0.0219 (3)	0.0744 (2)	0.0397 (6)	
C6	0.2311 (4)	0.1466 (3)	0.0483 (2)	0.0383 (6)	
H6A	0.2122	0.1962	−0.0291	0.046*	
C7	0.2217 (4)	0.2382 (3)	0.1391 (2)	0.0356 (6)	
C8	0.2483 (4)	0.1712 (3)	0.2565 (2)	0.0394 (6)	
C9	0.2896 (4)	0.0022 (3)	0.2777 (2)	0.0461 (7)	
H9A	0.3106	−0.0474	0.3548	0.055*	
C10	0.3010 (4)	−0.0948 (3)	0.1907 (2)	0.0456 (7)	
C11	0.3466 (7)	−0.2777 (4)	0.2205 (3)	0.0822 (13)	
H11A	0.3653	−0.3065	0.3035	0.123*	
H11B	0.4607	−0.3358	0.1691	0.123*	
H11C	0.2428	−0.3069	0.2079	0.123*	
C12	0.2353 (4)	0.2736 (3)	0.3534 (2)	0.0402 (6)	
C13	0.2136 (6)	0.2797 (4)	0.5634 (3)	0.0723 (11)	
H13A	0.2095	0.2093	0.6370	0.108*	
H13B	0.0999	0.3754	0.5674	0.108*	
H13C	0.3230	0.3125	0.5531	0.108*	

### Atomic displacement parameters (Å^2^)

**Table:**

	*U*^11^	*U*^22^	*U*^33^	*U*^12^	*U*^13^	*U*^23^
N1	0.0804 (19)	0.0346 (12)	0.0354 (12)	−0.0212 (12)	−0.0131 (12)	−0.0035 (9)
O1	0.165 (3)	0.0436 (13)	0.0494 (13)	−0.0360 (15)	−0.0326 (15)	0.0018 (10)
C1	0.090 (3)	0.072 (2)	0.0495 (19)	−0.032 (2)	−0.0078 (18)	−0.0197 (16)
N2	0.0652 (17)	0.0380 (13)	0.0380 (13)	−0.0210 (13)	−0.0154 (12)	−0.0009 (10)
O2	0.0695 (16)	0.0383 (12)	0.0819 (17)	0.0019 (11)	−0.0134 (13)	−0.0029 (11)
C2	0.059 (2)	0.0473 (16)	0.0487 (17)	−0.0144 (14)	−0.0101 (15)	−0.0114 (13)
O3	0.0922 (18)	0.0483 (12)	0.0568 (13)	−0.0405 (12)	−0.0019 (12)	0.0022 (10)
C3	0.0543 (18)	0.0422 (15)	0.0443 (16)	−0.0216 (13)	−0.0042 (13)	−0.0099 (12)
O4	0.1073 (18)	0.0430 (11)	0.0345 (11)	−0.0323 (11)	−0.0149 (11)	−0.0013 (9)
C4	0.0537 (18)	0.0375 (15)	0.0423 (16)	−0.0169 (13)	−0.0060 (13)	−0.0029 (12)
O5	0.1011 (18)	0.0393 (11)	0.0447 (12)	−0.0343 (11)	−0.0136 (11)	−0.0036 (9)
C5	0.0521 (17)	0.0328 (13)	0.0365 (14)	−0.0161 (12)	−0.0063 (12)	−0.0072 (11)
C6	0.0496 (17)	0.0333 (13)	0.0312 (13)	−0.0133 (12)	−0.0063 (12)	−0.0035 (10)
C7	0.0435 (16)	0.0272 (12)	0.0377 (14)	−0.0140 (11)	−0.0081 (12)	−0.0007 (10)
C8	0.0469 (17)	0.0336 (13)	0.0402 (15)	−0.0168 (12)	−0.0065 (12)	−0.0036 (11)
C9	0.071 (2)	0.0356 (14)	0.0319 (14)	−0.0193 (14)	−0.0096 (13)	0.0002 (11)
C10	0.070 (2)	0.0306 (13)	0.0362 (14)	−0.0193 (13)	−0.0060 (13)	−0.0003 (11)
C11	0.164 (4)	0.0358 (16)	0.0499 (19)	−0.035 (2)	−0.026 (2)	0.0017 (14)
C12	0.0459 (17)	0.0356 (14)	0.0384 (15)	−0.0138 (12)	−0.0063 (12)	−0.0028 (11)
C13	0.129 (3)	0.0519 (19)	0.0386 (18)	−0.032 (2)	−0.0169 (19)	−0.0070 (14)

### Geometric parameters (Å, °)

**Table:**

N1—C4	1.361 (3)	O4—C13	1.446 (3)
N1—C5	1.398 (3)	O5—C12	1.190 (3)
N1—H1A	0.8600	C5—C6	1.394 (3)
O1—C4	1.202 (3)	C5—C10	1.399 (4)
C1—C2	1.524 (4)	C6—C7	1.377 (3)
C1—H1B	0.9600	C6—H6A	0.9300
C1—H1C	0.9600	C7—C8	1.391 (3)
C1—H1D	0.9600	C8—C9	1.391 (4)
N2—O3	1.218 (3)	C8—C12	1.490 (4)
N2—O2	1.218 (3)	C9—C10	1.370 (4)
N2—C7	1.474 (3)	C9—H9A	0.9300
C2—C3	1.516 (4)	C10—C11	1.512 (4)
C2—H2A	0.9700	C11—H11A	0.9600
C2—H2B	0.9700	C11—H11B	0.9600
C3—C4	1.507 (4)	C11—H11C	0.9600
C3—H3A	0.9700	C13—H13A	0.9600
C3—H3B	0.9700	C13—H13B	0.9600
O4—C12	1.327 (3)	C13—H13C	0.9600
			
C4—N1—C5	129.3 (2)	C7—C6—C5	118.9 (2)
C4—N1—H1A	115.4	C7—C6—H6A	120.6
C5—N1—H1A	115.4	C5—C6—H6A	120.6
C2—C1—H1B	109.5	C6—C7—C8	123.3 (2)
C2—C1—H1C	109.5	C6—C7—N2	115.7 (2)
H1B—C1—H1C	109.5	C8—C7—N2	121.0 (2)
C2—C1—H1D	109.5	C7—C8—C9	115.8 (2)
H1B—C1—H1D	109.5	C7—C8—C12	122.1 (2)
H1C—C1—H1D	109.5	C9—C8—C12	122.1 (2)
O3—N2—O2	124.3 (2)	C10—C9—C8	123.4 (3)
O3—N2—C7	117.5 (2)	C10—C9—H9A	118.3
O2—N2—C7	118.1 (2)	C8—C9—H9A	118.3
C3—C2—C1	112.0 (3)	C9—C10—C5	119.0 (2)
C3—C2—H2A	109.2	C9—C10—C11	120.3 (2)
C1—C2—H2A	109.2	C5—C10—C11	120.7 (2)
C3—C2—H2B	109.2	C10—C11—H11A	109.5
C1—C2—H2B	109.2	C10—C11—H11B	109.5
H2A—C2—H2B	107.9	H11A—C11—H11B	109.5
C4—C3—C2	113.6 (2)	C10—C11—H11C	109.5
C4—C3—H3A	108.8	H11A—C11—H11C	109.5
C2—C3—H3A	108.8	H11B—C11—H11C	109.5
C4—C3—H3B	108.8	O5—C12—O4	123.6 (2)
C2—C3—H3B	108.8	O5—C12—C8	124.7 (2)
H3A—C3—H3B	107.7	O4—C12—C8	111.7 (2)
C12—O4—C13	116.5 (2)	O4—C13—H13A	109.5
O1—C4—N1	122.4 (3)	O4—C13—H13B	109.5
O1—C4—C3	123.6 (3)	H13A—C13—H13B	109.5
N1—C4—C3	114.0 (2)	O4—C13—H13C	109.5
C6—C5—N1	122.0 (2)	H13A—C13—H13C	109.5
C6—C5—C10	119.7 (2)	H13B—C13—H13C	109.5
N1—C5—C10	118.3 (2)		
			
C1—C2—C3—C4	−178.2 (3)	C6—C7—C8—C12	−179.3 (3)
C5—N1—C4—O1	−3.1 (5)	N2—C7—C8—C12	−1.3 (4)
C5—N1—C4—C3	177.3 (3)	C7—C8—C9—C10	−1.1 (4)
C2—C3—C4—O1	1.4 (4)	C12—C8—C9—C10	179.5 (3)
C2—C3—C4—N1	−179.1 (3)	C8—C9—C10—C5	−0.1 (5)
C4—N1—C5—C6	0.1 (5)	C8—C9—C10—C11	180.0 (3)
C4—N1—C5—C10	179.8 (3)	C6—C5—C10—C9	1.3 (4)
N1—C5—C6—C7	178.5 (3)	N1—C5—C10—C9	−178.4 (3)
C10—C5—C6—C7	−1.2 (4)	C6—C5—C10—C11	−178.8 (3)
C5—C6—C7—C8	−0.1 (4)	N1—C5—C10—C11	1.5 (4)
C5—C6—C7—N2	−178.3 (2)	C13—O4—C12—O5	1.0 (4)
O3—N2—C7—C6	−75.9 (3)	C13—O4—C12—C8	179.9 (3)
O2—N2—C7—C6	101.7 (3)	C7—C8—C12—O5	−13.9 (4)
O3—N2—C7—C8	105.9 (3)	C9—C8—C12—O5	165.5 (3)
O2—N2—C7—C8	−76.5 (3)	C7—C8—C12—O4	167.1 (3)
C6—C7—C8—C9	1.2 (4)	C9—C8—C12—O4	−13.5 (4)
N2—C7—C8—C9	179.3 (3)		

### Hydrogen-bond geometry (Å, °)

**Table:**

*D*—H···*A*	*D*—H	H···*A*	*D*···*A*	*D*—H···*A*
C6—H6A···O1	0.93	2.20	2.809 (3)	122
C9—H9A···O4	0.93	2.41	2.734 (3)	100
C13—H13A···O1^i^	0.96	2.33	3.284 (4)	174

Symmetry codes: (i) *x*, *y*, *z*+1.
